# Study on the Motion Trajectory of Abrasives and Surface Improvement Mechanism in Ultrasonic-Assisted Diamond Wire Sawing Monocrystalline Silicon

**DOI:** 10.3390/mi16060708

**Published:** 2025-06-13

**Authors:** Honghao Li, Yufei Gao, Shengtan Hu, Zhipu Huo

**Affiliations:** 1 Key Laboratory of High Efficiency and Clean Mechanical Manufacture of Ministry of Education, School of Mechanical Engineering, Shandong University, Jinan 250061, China; 2 State Key Laboratory of Advanced Equipment and Technology for Metal Forming, Shandong University, Jinan 250061, China; 3 Shandong Key Laboratory of High Performance Tools and System, Shandong University, Jinan 250061, China

**Keywords:** diamond wire saw, surface morphology, ultrasonic vibration, wire marks

## Abstract

The surface quality of diamond wire sawing (DWS) wafers directly affects the efficiency and yield of subsequent processing steps. This paper investigates the motion trajectory of abrasives in ultrasonic-assisted diamond wire sawing (UADWS) and its mechanism for improving surface quality. The influence of ultrasonic vibration on the cutting arc length, cutting depth, and interference of multi-abrasive trajectories was analyzed through the establishment of an abrasive motion trajectory model. The ultrasonic vibration transforms the abrasive trajectory from linear to sinusoidal, thereby increasing the cutting arc length while reducing the cutting depth. A lower wire speed was found to be more conducive to exploiting the advantages of ultrasonic vibration. Furthermore, the intersecting interference of multi-abrasive trajectories contributes to enhanced surface quality. Experimental studies were conducted on monocrystalline silicon (mono-Si) to verify the effectiveness of ultrasonic vibration in improving surface morphology and reducing wire marks during the sawing process. The experimental results demonstrate that, compared with DWS, UADWS achieves a significantly lower surface roughness *Ra* and generates micro-pits. The ultrasonic vibration induces a micro-grinding effect on both peaks and valleys of wire marks, effectively reducing their peak–valley (PV) height. This study provides a theoretical basis for optimizing UADWS process parameters and holds significant implications for improving surface quality in mono-Si wafer slicing.

## 1. Introduction

As a crucial energy form, solar energy plays a pivotal role in optimizing energy structures through its development and utilization [1,2,3]. In photovoltaic technology, mono-Si solar cells dominate the global solar market [4,5], owing to their high photoelectric conversion efficiency [6]. Slicing processing is the first process in the manufacturing process of monocrystalline silicon solar cell substrates, and the surface quality has an important influence on the subsequent processes [7]. With the advancement of manufacturing technology, researchers have developed UADWS technology based on DWS.

When ultrasonic vibration is applied to the wire, one of the important differences compared to sawing by a DWS is that the motion trajectory of the abrasives in the processing area changes, which further causes changes in the sawing force and surface morphology [8]. Therefore, exploring the changes in the abrasive motion trajectories in the processing area is of great significance for in-depth research on the mechanism by which UADWS technology improves the surface quality of workpieces. Wang et al. [9] investigated the influence of the motion trajectory of abrasives during the sawing of mono-Si by UADWS on the formation of the surface morphology of silicon wafers. The research first established the surface model of the diamond wire through the equal probability method. On this basis, the motion trajectory equation of the abrasives in the processing area after applying ultrasonic vibration to the wire was derived. The research finds that the motion trajectory of abrasives is affected by parameters such as wire speed, feed speed, and workpiece rotational speed. Finally, the influence of the variation in the abrasive motion trajectory on the surface morphology and surface roughness was verified through experiments. This research provides a theoretical basis for optimizing sawing parameters and understanding the formation mechanism of the surface morphology of mono-Si slices. Qin et al. [10] established the distribution position and morphology of abrasives on the grinding wheel surface. On this basis, they established the motion trajectory of abrasives on the grinding wheel surface in the ultrasonic-assisted grinding processing area and conducted simulation analysis on the motion trajectories of different abrasives. Through the SiC grinding experiment, it was verified that after applying ultrasonic vibration to the grinding wheel, the movement of abrasives could repeatedly process the same position on the workpiece surface, thereby improving the surface quality. Compared with ordinary processing, the surface roughness of the workpiece was reduced by 16.7% to 32.6% after applying ultrasonic vibration. Although this research focused on ultrasonic-assisted grinding, the analysis of the abrasive motion trajectory in the processing area and the influence mechanism of ultrasonic vibration on the processing process have important reference value for the research of UADWS technology.

Slicing, as one of the important processing procedures in material application, requires a relatively high surface quality to reduce the subsequent processing cost [11,12]. Therefore, scholars have conducted extensive research on the surface quality of slices cut by UADWS. Through comparative experiments, Li et al. [13] systematically studied the surface quality of single-crystal SiC slices cut by DWS and UADWS. The results show that, compared with DWS, UADWS can reduce the surface roughness *Ra* of the slices by 26% to 55%. Yan et al. [14] found that when sawing composite ceramics with UADWS, due to the effect of ultrasonic vibration, the wire saw could cut the fibers smoothly and reduce the pits on the surface of the slices. And after the application of ultrasonic vibration, the surface roughness *Ra* of the slice is lower. Li et al. [15] investigated the influence of processing parameters such as wire speed, workpiece feed speed, workpiece rotational speed, and ultrasonic amplitude on the surface roughness of the slice. Increasing the ultrasonic amplitude can reduce the surface roughness of the material. With an increase in the wire speed, the surface roughness of the slice will also decrease. Wang et al. [16] used DWS and UADWS to cut mono-Si workpieces and found that the surface roughness was higher with DWS, with larger pits and obvious scratches. The UADWS-cut slices had a lower roughness and smaller pits. When evaluating surface quality in diamond wire sawing, assessment criteria extend beyond surface roughness, as wire marks constitute another critical parameter that significantly influences the evaluation outcome [17,18]. Periodically spaced wire marks are distinctly visible on diamond wire-sawn wafers [19]. The reciprocating motion of the wire saw combined with guide wheel installation errors induces cyclic lateral forces during processing, causing periodic wire deflection within the kerf that generates wavy-patterned wire marks on the wafer surface [20]. These surface marks not only elevate the risk of silicon wafer fracture but also adversely affect subsequent manufacturing processes [21]. Li et al. [22] experimentally demonstrated that the workpiece reciprocating movement-assisted diamond wire sawing (RMA-DWS) of Si_3_N_4_ ceramics yielded slices with reduced wire marks and lower PV values compared to DWS. These findings suggest that modifying the relative motion between the wire and workpiece in the processing area may beneficially influence wire mark formation. Xu et al. [23] employed UADWS to cut NdFeB and investigate the formation mechanism of wire marks. The results indicate that when the feed speed is increased to 0.2 mm/min, ultrasonic vibration can play a better role. The minute vibrations of abrasives can generate a micro-grinding effect on the surface of the sliced samples, thereby improving the surface morphology and reducing the PV value of wire marks. The aforementioned scholars have experimentally demonstrated that UADWS can enhance the surface roughness and mitigate wire marks of slices. However, most of their analyses on the surface improvement mechanisms after introducing ultrasonic vibration are conducted from a macroscopic perspective. The most crucial aspect of adding ultrasonic vibration lies in its alteration of the abrasives’ motion states and cutting trajectories, which in turn exert a beneficial influence on the sliced surface. Therefore, it is of great significance to analyze the microscopic motion states of abrasives after applying ultrasonic vibration to the wire saw and to investigate how the changes in abrasive motion affect the improvement of the sliced surface. This will further elucidate the processing mechanism of UADWS.

**Figure 1:**
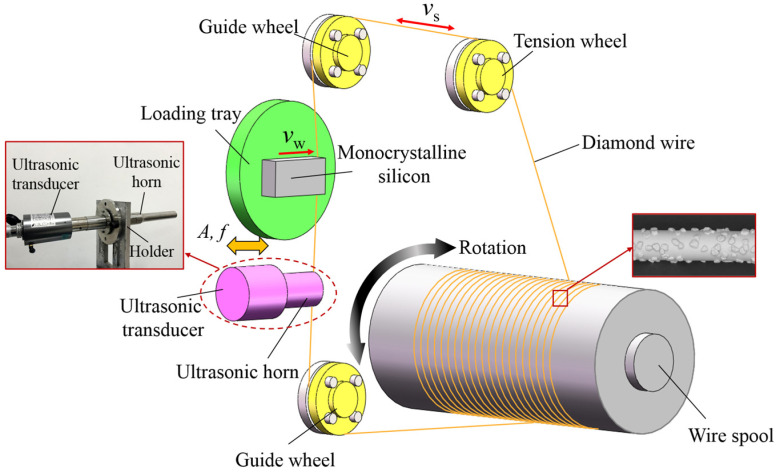
Schematic diagram of UADWS processing.

The processing principle of the UADWS is shown in Figure 1. The entire processing system consists of a wire saw cutting machine tool and an ultrasonic vibration system. The diamond wire saw is wound on the wire cylinder. During processing, the tension wheel tensifies the wire saw with a constant tension force *F*_t_. The motor drives the reel to rotate, and the reel drives the wire saw to reciprocate at a speed v_s_. The workpiece is fed at a speed *v*_w_. Ultrasonic vibration is applied to the wire saw through the ultrasonic horn. While the wire saw moves back and forth to cut, high-frequency vibration is generated parallel to the feed direction of the workpiece, achieving the removal of the material. In this paper, the motion trajectories of abrasives during UADWS are analyzed. Combined with experiments, the mechanism by which ultrasonic vibration improves the surface roughness and wire marks of slices is revealed from a microscopic perspective.

The structure of the paper is as follows. In Section 2, the motion trajectories, cutting arc lengths, and cutting depths of the abrasives are analyzed. In Section 3, the experimental materials and schemes are introduced. In Section 4, based on the experimental results, a comparative analysis is conducted on the differences in the surface morphologies of slices obtained by DWS and UADWS. Finally, conclusions are drawn in Section 5.

## 2. Kinematic Analysis of Abrasives in UADWS

### 2.1. Analysis of Abrasive Motion Trajectories

**Figure 2:**
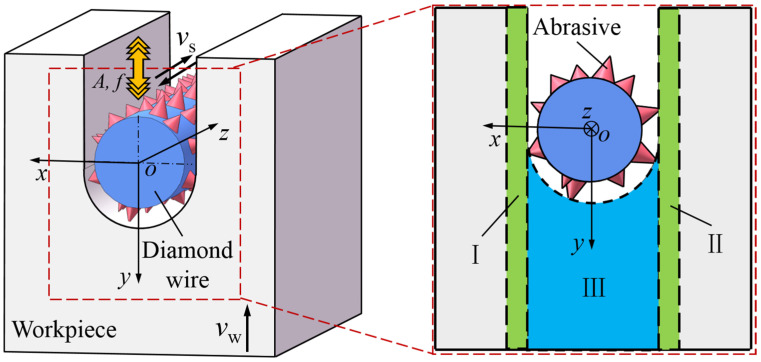
Schematic diagram of the processing area.

Due to the size of the workpiece or the limitations of the working platform, ultrasonic vibration excitation is usually applied to the wire saw. The schematic diagram of the cutting processing area of the UADWS is shown in Figure 2. Taking the center of the cross-section of the wire saw as the origin, an *O-xyz* rectangular coordinate system as shown in the figure is established. The wire saw generates high-frequency ultrasonic vibration with an amplitude of *A* and a frequency of *f* along the *y*-axis, that is, the feed direction of the workpiece. The feed speed of the workpiece is *v*_w_. Under the action of the driving roller, the wire saw moves back and forth along the *z*-axis at a speed of *v*_s_. When the wire saw removes material in the cutting kerf, the abrasives at the bottom of the wire saw come into intermittent contact with the workpiece due to the effect of ultrasonic vibration. The abrasives on both sides of the wire saw interact with the material and eventually form the surface of the slice. The ultrasonic vibration effect has changed the movement trajectories of the abrasives on both sides. Therefore, the following will conduct a specific analysis based on the movement of the abrasives.

#### 2.1.1. Establishment of the Motion Equation of Single Abrasive

As can be seen from Figure 2, in the wire saw processing area, areas I and II on both sides of the cutting kerf are formed by the interaction of abrasives with the workpiece to create the surface of the slice, while the material in area III is formed by the removal of abrasives from the bottom of the wire saw to create the kerf. After applying ultrasonic vibration, the change in the movement mode of abrasives on both sides of the wire saw will affect the surface quality of the workpiece. Therefore, the abrasives in this part of the area are analyzed. Since the distribution of abrasives on the surface of the wire saw is random [24,25], in order to simplify the calculation process, only the abrasives on one side of the wire saw are taken for analysis to establish the motion equation of the abrasives.

**Figure 3:**
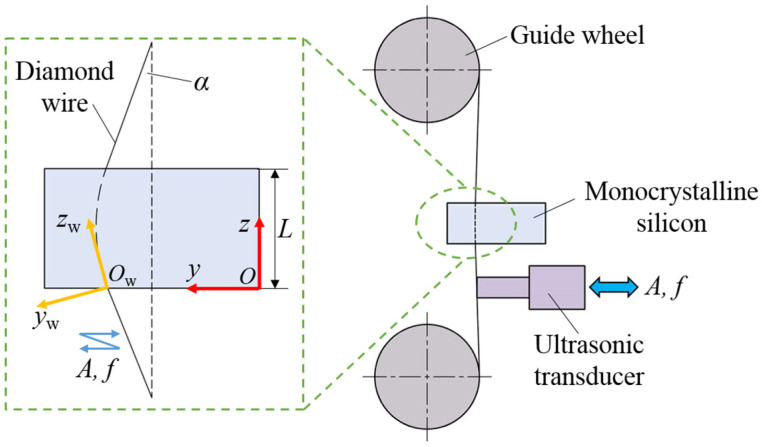
Schematic diagram of the UADWS.

In order to conduct an in-depth study on the differences between ultrasonic vibration abrasive trajectories and ordinary abrasive trajectories, it is necessary to establish the abrasive motion equations for UADWS and DWS, respectively. Figure 2 shows the cutting model of the ultrasonic vibration-assisted processing area. The workpiece is fed at the speed *v*_w_, and ultrasonic vibration with frequency *f* and amplitude *A* is applied to the wire saw. During the processing, the wire saw will bend to form a wire bow [26,27] with angle *α*. The coordinate system *O*-*zy* for the motion of the workpiece relative to the machine tool and the coordinate system *O*_w_-*z*_w_*y*_w_ for the motion of the abrasives relative to the workpiece are established, as shown in Figure 3.

At time *t*, in the coordinate system *O*-*zy*, the motion of the workpiece in the *y* direction can be expressed as

(1) $$y(t)=v_{w}t$$

where *v*_w_ represents the feed speed; *t* represents the movement time.

The movement of the workpiece in the *z* direction can be expressed as

(2) z(t)=0

It can be seen from Equations (1) and (2) that in the coordinate system *O*-*zy*, the motion trajectory of the workpiece can be expressed as

(3) $$\left\{\begin{array}{l} y(t)=v_{w}t\\ z(t)=0 \end{array}\right.$$

Since the wire saw bends during the sawing process to produce a wire bow, the motion trajectory of the workpiece is coordinate-transformed according to the two-dimensional coordinate rotation rule, which can be expressed as

(4) $$\left[\begin{array}{c} z_{w}(t)\\ y_{w}(t) \end{array}\right]=\left[\begin{array}{cc} \cos\alpha & \sin\alpha \\ -\sin\alpha & \cos\alpha \end{array}\right]\left[\begin{array}{c} z(t)\\ y(t) \end{array}\right]$$

where *α* is the angle of the wire bow.

Substituting Equation (3) into Equation (4), the motion trajectory of the workpiece in the coordinate system *O*_w_-*z*_w_*y*_w_ can be expressed as

(5) $$\left\{\begin{array}{l} z_{w}(t)=v_{w}\cdot t\cdot \sin\alpha \\ y_{w}(t)=v_{w}\cdot t\cdot \cos\alpha \end{array}\right.$$

When ultrasonic vibration is applied to the wire saw, the equation can be expressed as follows [28]:

(6) U(t)=Asin(ωt+φ)

where *A* represents the ultrasonic amplitude; *ω* = 2π*f*; *f* is the ultrasonic vibration frequency; φ is the phase angle.

The abrasives are consolidated on the wire saw substrate by electroplating or other means. Therefore, it can be considered that the movement law is the same as that of the wire saw. The component of the ultrasonic vibration abrasives in the *z*_w_ direction can be expressed as

(7) $$z_{a}(t)=A\sin(\omega t+\varphi )\cdot \sin\alpha$$

The component in the *y*_w_ direction can be expressed as

(8) $$y_{a}(t)=A\sin(\omega t+\varphi )\cdot \cos\alpha$$

The wire saw itself has axial movement during the processing; therefore, in the *O*_w_-*z_w_y_w_* coordinate system, the movement of the abrasives in the *z*_w_ direction can be expressed as

(9) $$z^{\prime }(t)=v_{s}t+A\sin(\omega t+\varphi )\cdot \sin\alpha$$

where *v*_s_ represents the wire speed.

Combining Equations (7)–(9), based on the principle of motion composition, it can be finally obtained that when ultrasonic vibration is applied to the wire saw, the motion trajectory of the abrasives in the processing area relative to the workpiece can be expressed in *O*_w_-*z_w_y_w_* as

(10) $$\left\{\begin{array}{l} z_{\mathrm{ua}}(t)=v_{s}t+A\sin(\omega t+\varphi )\cdot \sin\alpha +v_{w}\cdot t\cdot \sin\alpha \\ y_{\mathrm{ua}}(t)=A\sin(\omega t+\varphi )\cdot \cos\alpha -v_{w}\cdot t\cdot \cos\alpha \end{array}\right.$$

When no ultrasonic vibration is applied to the wire saw, that is, when cutting with DWS, the motion trajectory of the abrasives relative to the workpiece is

(11) $$\left\{\begin{array}{l} z_{\mathrm{ca}}(t)=v_{s}t+v_{w}\cdot t\cdot \sin\alpha \\ y_{\mathrm{ca}}(t)=-v_{w}\cdot t\cdot \cos\alpha \end{array}\right.$$

By comparing the motion equations of the abrasives in the two processing methods above, it can be seen that due to the application of ultrasonic vibration to the wire saw in the feed direction of the workpiece, the motion characteristics of the abrasives have changed. Therefore, it is necessary to further analyze the motion of the abrasives.

#### 2.1.2. Analysis of the Motion Trajectory of Single Abrasive

In the UADWS, the introduction of ultrasonic vibration changes the cutting trajectory of the abrasives. After obtaining the time for the abrasives to process the workpiece, the motion trajectory of the abrasives can be visualized by using MATLAB (2021a) according to Equations (10) and (11).

Suppose the wire bow is symmetrical about the centerline of the workpiece. Take the side where the abrasive cuts into the workpiece for analysis. For a workpiece of length *L*, the cutting length of the abrasive equals *L*/2, and the time taken for the abrasive to traverse from the entry to the exit of the workpiece is denoted as Δ*t*.

When the wire speed is *v*_s_, the time Δ*t* taken for abrasive cutting can be expressed as

(12) $$\Delta t=\frac{L}{2v_{s}}$$

**Figure 4:**
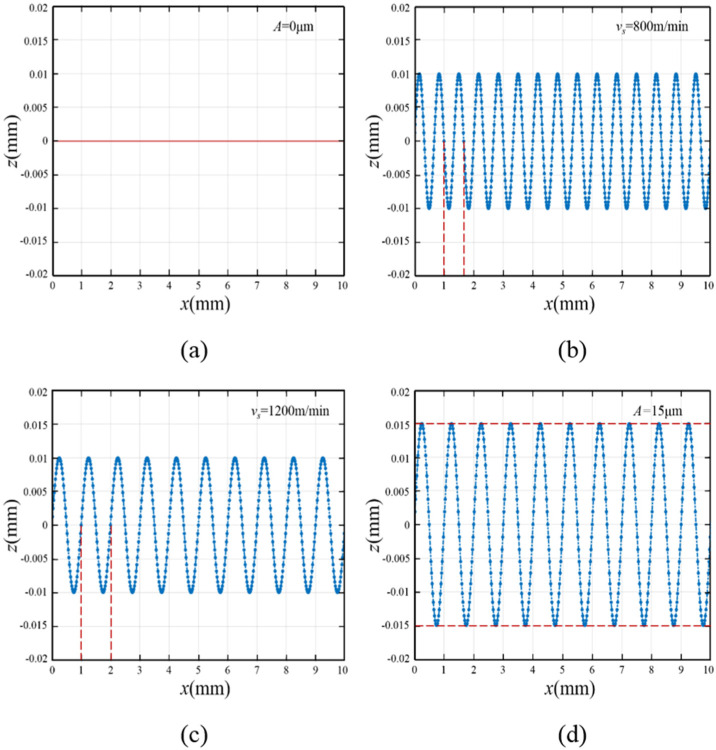
Simulation results of single abrasive motion trajectory under different processing conditions: (**a**) DWS; (**b**) *v*_s_ = 800 m/min, *A* = 10 μm; (**c**) *v*_s_ = 1200 m/min, *A* = 10 μm; (**d**) *v*_s_ = 1200 m/min, *A* = 15 μm.

According to Equations (10)–(12), the cutting trajectory of a single abrasive in the processing area can be simulated and calculated by using MATLAB. According to the experimental conditions, the processing parameters were set. When the feed speed *v*_w_ = 0.7 mm/min, the workpiece processing length *L* = 20 mm, and the ultrasonic frequency *f* = 20 kHz, the movement trajectories of a single abrasive during conventional cutting and ultrasonic cutting are shown in Figure 4.

Figure 4a,b show the abrasive motion trajectories of DWS and UADWS under the same wire speed. When no ultrasonic vibration is applied to the abrasives, the feed speed of the workpiece is very small relative to the wire speed, and thus the displacement of the abrasives in the *y* direction is extremely small. It can be seen from the simulation results that the cutting trajectory of the abrasives during processing is approximately a straight line. When ultrasonic vibration is applied, the abrasives perform high-frequency vibration cutting in the processing area. When the workpiece lengths are the same, the cutting length of the abrasives on the workpiece surface during UADWS is greater than that in DWS, which increases the material removal amount.

When other processing parameters are the same, it can be obtained from Equation (12) that the wire speed determines the time for a single abrasive to cut the workpiece in the processing area. When the wire speed is large, the abrasive will leave the processing area rapidly. As can be seen from the dotted lines in Figure 4b,c, when the wire speed increases, the abrasives vibrate for one cycle in the processing area, and the distance they move in the *x*-axis direction will increase. That is, within the same period of time, when the wire speed increases, the wavelength of the abrasive’s movement trajectory will increase. Therefore, when the abrasive is cut within the processing area, the number of vibrations will be reduced. Compared with a low wire speed, a high wire speed will result in fewer complete sinusoidal waveform trajectories in the processing area, thereby weakening the effect of ultrasonic vibration to a certain extent.

Figure 4c, d show the motion trajectories of abrasives in the processing area when other processing parameters are the same but different ultrasonic amplitudes are used for processing. As can be seen from Figure 4d, when the ultrasonic amplitude increases, the movement length of the abrasives in the *z*-axis direction will increase, thereby increasing the cutting trajectory length of the abrasives. Furthermore, the increase in ultrasonic amplitude makes it easier for the trajectories of different abrasives to cross, enhancing the interference degree of the trajectories of different abrasives. There is a mutual scraping effect among the abrasive trajectories, which is helpful to improve the surface quality of the workpiece.

**Figure 5:**
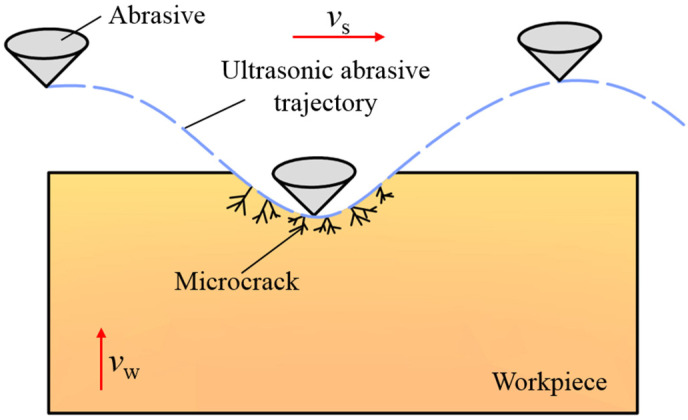
Schematic diagram of the motion trajectory of the abrasives at the bottom of UADWS.

The abrasives at the bottom of the wire saw mainly function to remove the material and form the cut kerf. Ultrasonic vibration also changes the contact mode between the abrasives at the bottom of the wire saw and the workpiece. As shown in Figure 5, when ultrasonic vibration is applied along the feed direction of the workpiece, the way the wire saw removes material from the workpiece changes from continuous contact to a high-frequency contact-separation mode. Moreover, due to the ultrasonic effect, the abrasives have a relatively high impact speed. Under the impact of the abrasives, microcracks can occur on the surface of the workpiece. When the abrasives cut this area subsequently, a smaller force can be used to remove the material in this area. When cutting workpieces, the interaction force generated when the abrasives at the bottom of the wire saw come into contact with the workpiece accounts for a large proportion of the macroscopic sawing force. Therefore, the wear of the abrasives is mostly concentrated in this area. The intermittent contact between the abrasives and the workpiece reduces the average sawing force during wire sawing, thereby reducing the wear of the wire. Moreover, it enables the coolant to effectively enter the processing area, reduce the sawing temperature, remove the chips and broken abrasives, and prevent secondary scratches on the surface of the workpiece.

In conclusion, the wire speed will affect the movement time of the abrasives in the processing area, thereby further influencing the vibration frequency and cutting trajectory length of the abrasives in the processing area. A lower wire speed is more conducive to giving full play to the role of ultrasonic vibration. The influence of ultrasonic amplitude on the abrasive trajectory is mainly reflected in the length of the abrasive cutting trajectory and the interference degree between different abrasive cutting trajectories. Specifically, when the ultrasonic amplitude increases, the movement length of the abrasives in the *z*-axis direction will increase, and the cutting trajectories of different abrasives are more likely to intersect, which can further remove surface defects and improve surface quality. Furthermore, the contact mode between the abrasives at the bottom of the wire saw and the workpiece changes from continuous contact to high-frequency intermittent contact, which can reduce the average sawing force, slow down the wear of the abrasives, and reduce surface damage.

#### 2.1.3. Analysis of the Motion Trajectories of Multiple Abrasives

According to the analysis in Section 2.1.2, when the ultrasonic vibration-assisted wire saw is cutting, the motion trajectory of the abrasives in the processing area is approximately a sine curve. Therefore, the abrasive trajectories at different positions on the wire saw surface will show cross-interference. According to the relative motion relationship between the abrasives and the workpiece, the wavelength of the sinusoidal motion trajectory of the abrasives is calculated as

(13) $$\lambda =v_{s}T=\frac{v_{s}}{f}$$

where *T* represents the ultrasonic vibration period; *f* is the ultrasonic vibration frequency.

**Figure 6:**
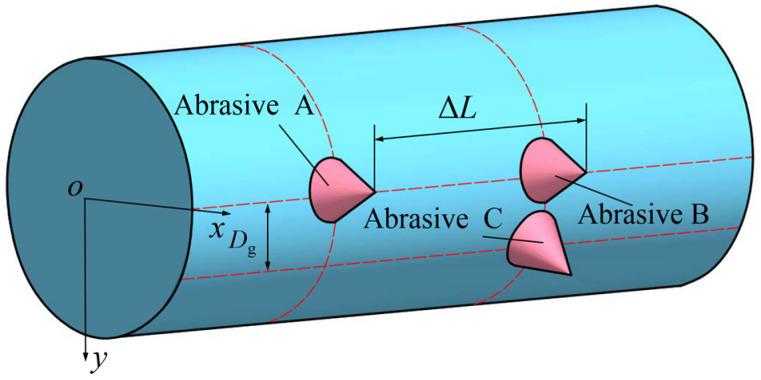
Schematic diagram of the distribution of adjacent abrasives on the wire saw surface.

It can be seen from Figure 4b,c that when *v*_s_ takes different values, the wavelengths of the abrasive cutting trajectories are also different. Therefore, the mutual interference among the abrasive movement trajectories at different positions on the wire saw surface is also different. Due to the mutual interference among the abrasive trajectories, the cutting trajectory of a single abrasive will be truncated by other abrasives. As shown in Figure 6, a section of the wire saw was taken to analyze the distribution of surface abrasives.

If the wire matrix is regarded as a cylinder, there are two situations regarding the distribution of adjacent abrasives. When the surface abrasives are located on the same generatrix but on different circles, as shown for abrasive A and abrasive B in Figure 6, assuming the distance between the abrasives is Δ*L*, when the distance Δ*L* between the adjacent abrasives takes different values, the interference of the motion trajectories of abrasive A and abrasive B is different.

**Figure 7:**
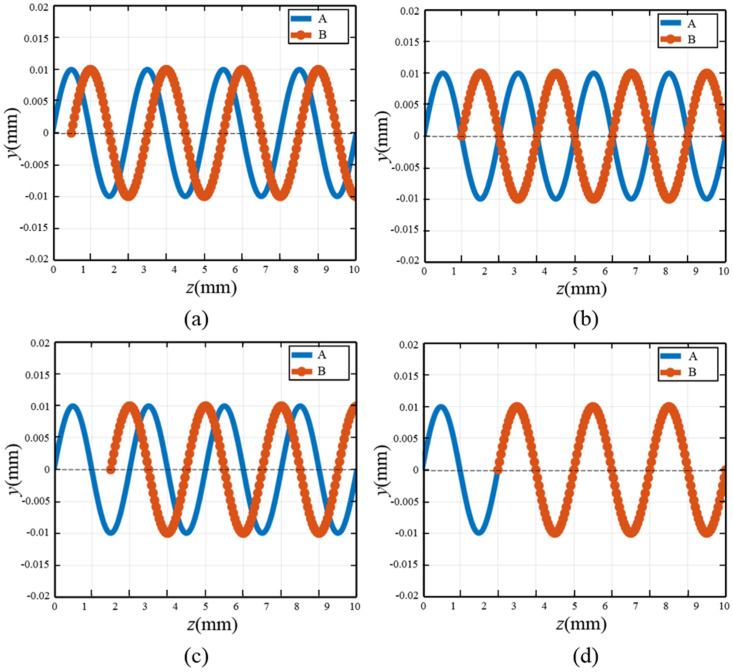
Interference conditions of adjacent abrasives on different circumferences: (**a**) Δ*L* = *λ*/4; (**b**) Δ*L* = *λ*/2; (**c**) Δ*L* = 3*λ*/4; (**d**) Δ*L* = *λ*.

Figure 7 shows the interference of the movement trajectories of abrasive A and abrasive B when cutting the workpiece surface under different relationships of Δ*L* and *λ*. When the degree of mutual interference of the abrasive trajectories is greater, the scratching effect of adjacent abrasives on surface scratches, sharp peaks, and other defects is more obvious, which can improve the surface quality to a certain extent, as shown in Figure 7a,c. When the interference degree between the abrasive trajectories is low, since the trajectories basically do not cross and overlap, the amount of material removed by the abrasives from the workpiece increases, improving the material removal rate, as shown in Figure 7b. However, if the intersection degree of the trajectories between adjacent abrasives is too large, or even if the movement trajectories overlap, it will reduce the removal rate of the material. As shown in Figure 7d, the subsequent movement trajectory of abrasive A coincides with abrasive B, which will significantly reduce the material removal amount of abrasive A.

**Figure 8:**
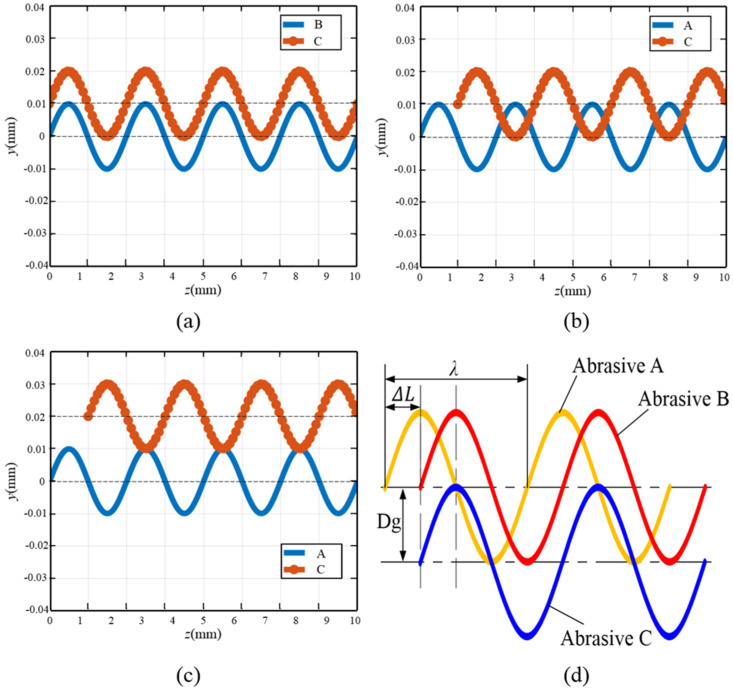
Interference conditions of abrasive trajectories with different generatrices. (**a**) Same circumference. (**b**) *D*_g_ < 2*A*. (**c**) *D*_g_ > 2*A*. (**d**) Multiple abrasive trajectories cross.

When the abrasives on the surface of the wire saw substrate are located on different generatrices, the motion interference between the abrasives is also different. The abrasive interference between different generatrices is shown in Figure 8.

Figure 8a shows the situation where the abrasives are located on the same circumference but on different generatrices. Due to the application of ultrasonic vibration to the wire saw, the abrasives are fixed on the wire saw substrate by electroplating or resin bonding, etc. Therefore, the movement of the abrasives on the wire saw is synchronous. In this case, there is no interference phenomenon between the cutting trajectories of abrasive B and abrasive C on the workpiece surface. Figure 8b shows the relationship between abrasive A and abrasive C when the abrasives are located on different circumferences and different generatrices. *A* represents the ultrasonic amplitude. When *D*_g_ < 2*A*, interference occurs between the abrasive cutting trajectories, and the abrasive trajectories are staggered twice at one wavelength. Figure 8c shows the case where *D*_g_ > 2*A*. It can be seen that when the generatrices’ spacing is greater than twice the ultrasonic amplitude, no interference phenomenon will occur between the abrasive trajectories regardless of the relationship between Δ*L* and *λ*. Figure 8d shows the interference of the cutting trajectories of abrasives A, B, and C. When the generatrix spacing of the adjacent abrasives is less than twice the ultrasonic amplitude and Δ*L* is not an integer multiple of *λ*, the trajectories of the adjacent abrasives will interweave. The surface of the wire saw is electroplated with a large number of diamond abrasives. When sawing with DWS, since the wire saw does not vibrate, the cross-interference between the abrasives is not obvious. After applying ultrasonic vibration to the wire saw, the movement trajectory of the abrasives changes. The cutting trajectories of the abrasives at different positions are interwoven and complex, interfering with each other. They repeatedly scratch the surface of the workpiece, generating an effect similar to polishing, which helps to improve the surface quality of the workpiece.

### 2.2. Analysis of Abrasive Cutting Parameters

Based on the above analysis, after the addition of ultrasonic vibration, the motion characteristics and trajectories of the abrasives undergo significant changes, which also leads to changes in the cutting trajectory length and cutting depth of the abrasives in the processing area. The changes in the arc length and depth of the abrasives further affect the sawing force and the surface quality of the slices. Therefore, analyzing the changes in the abrasive cutting parameters after the addition of ultrasonic waves can provide a theoretical basis for the subsequent calculation of the sawing force and analysis of the surface quality of the slices.

#### 2.2.1. Abrasive Cutting Arc Length

In sawing processing, the cutting length of abrasives in the processing area is one of the important parameters. The cutting length not only affects the amount of material removed, but also influences the sawing force and the surface quality. Therefore, analyzing the variation in the cutting arc length of abrasives in the processing area during UADWS is of great significance for studying the mechanism of ultrasonic assistance in reducing sawing force and improving the surface quality of slices.

The length of the motion trajectory of abrasives on the workpiece surface is closely related to the processing parameters. By differentiating the trajectory equation of the abrasives with respect to time, the velocity equation of the abrasives in the processing area can be obtained. Therefore, the velocity equation of the abrasives in the processing area cut by UADWS can be expressed as

(14) $$\left\{\begin{array}{l} v_{\mathrm{uz}}(t)=v_{s}+A\omega \cos(\omega t+\varphi )\cdot \sin\alpha +v_{w}\cdot \sin\alpha \\ v_{\mathrm{uy}}(t)=A\omega \cos(\omega t+\varphi )\cdot \cos\alpha -v_{w}\cdot \cos\alpha \end{array}\right.$$

The velocity equation of abrasives cut by DWS in the processing area can be expressed as

(15) $$\left\{\begin{array}{l} v_{\mathrm{cz}}(t)=v_{s}+v_{w}\cdot \sin\alpha \\ v_{\mathrm{cy}}(t)=-v_{w}\cdot \cos\alpha \end{array}\right.$$

According to the arc length expression and the abrasive velocity equation, it can be obtained that when the UADWS cuts, the trajectory length of the abrasive in the processing area is

(16) $$l_{u}=\int_{0}^{\Delta t}\sqrt{{\left[v_{s}+A\omega \cos(\omega t+\varphi )\sin\alpha +v_{w}\sin\alpha \right]}^{2}+{\left[A\omega \cos(\omega t+\varphi )\cos\alpha -v_{w}\cos\alpha \right]}^{2}}dt$$

For DWS, the trajectory length of the abrasives in the processing area is

(17) $$l_{c}=\int_{0}^{\Delta t}\sqrt{{\left(v_{s}+v_{w}\cdot \sin\alpha \right)}^{2}+{\left(v_{w}\cdot \cos\alpha \right)}^{2}}dt$$

where Δ*t* = *L*/2*v*_s_.

By comparing the abrasive motion trajectories of UADWS and DWS, it can be seen that when the workpiece length is the same, after applying ultrasonic vibration to the wire saw, the length of the abrasive motion trajectory is greater than that of DWS.

**Figure 9:**
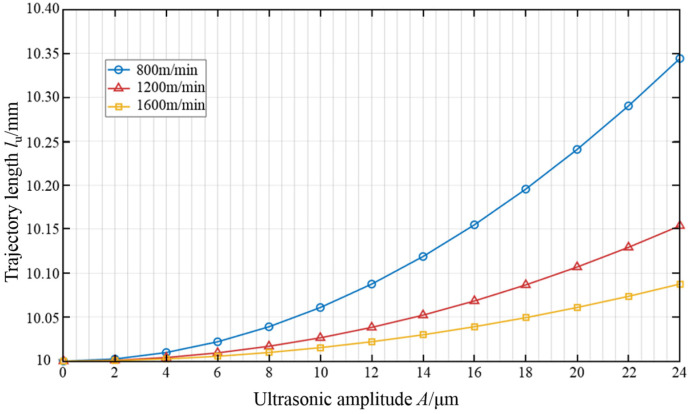
The influence of ultrasonic amplitude and wire speed on the length of abrasive cutting trajectory.

According to Equations (16) and (17), the influence of wire speed and ultrasonic amplitude on the length of abrasive cutting trajectory is mainly discussed when the ultrasonic frequency is constant. Figure 9 shows the influence of the ultrasonic amplitude and the wire speed on the length of the trajectory of a single abrasive in the processing area when the ultrasonic frequency is 20 kHz and the feed speed is 0.7 mm/min.

As can be seen from the Figure 9, when the amplitude is zero, that is, when no ultrasonic vibration is applied to the wire saw, the feed speed is very small compared to the wire speed. Therefore, the trajectory length of the abrasive in the processing area is approximately a straight line, that is, equal to the length of the workpiece. With the continuous increase in the ultrasonic amplitude, the length of the motion trajectory of the ultrasonic abrasives gradually increases. It can also be found that when the wire speed is relatively high, even if the ultrasonic amplitude is increased, the length of the movement trajectory of the ultrasonic abrasives is not much different from that of the ordinary abrasive cutting, and the change is not obvious. This is because when the wire speed increases, the abrasives will quickly leave the processing area. Therefore, the number of vibrations in the processing area decreases, and the effect of ultrasonic waves is not significant. When processing with a lower wire speed, the abrasives stay in the processing area for a longer time and the number of vibrations increases. Therefore, the length of the abrasive trajectory significantly increases. In order to increase the movement trajectory length of ultrasonic abrasives and enhance the improvement effect of ultrasonic vibration on processing, an appropriate wire speed and ultrasonic amplitude should be selected.

#### 2.2.2. Abrasive Cutting Depth

During processing, the cutting depth of the abrasives will affect the sawing force and the final surface quality. When the cutting depth of the abrasives is larger, the material is more prone to brittle removal, resulting in an increase in sawing force and a decrease in surface quality. Therefore, the variation in abrasive cutting depth after the application of ultrasonic vibration also needs to be explored. During the cutting process, the cutting depth of the abrasives on both sides of the wire saw has a significant impact on the final surface, while the abrasives at the bottom of the wire saw mainly remove the material to form a cut kerf. Therefore, this section discusses the abrasive cutting depth on both sides of the wire saw.

**Figure 10:**
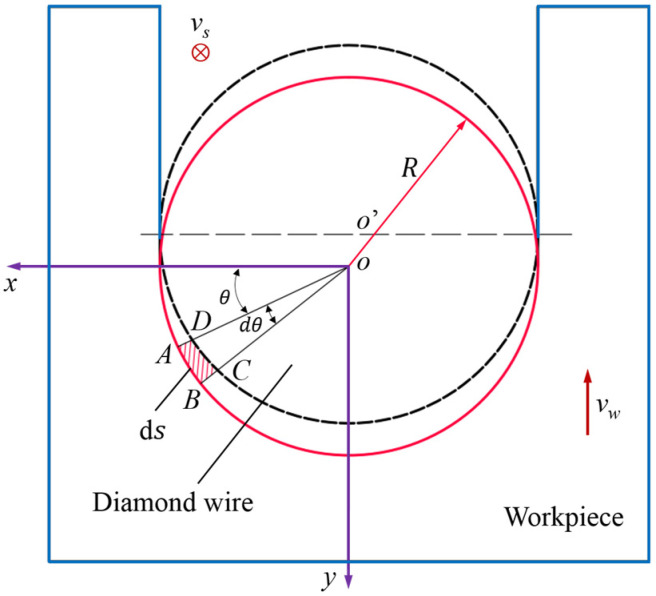
Schematic diagram of the cross-section of the wire sawing.

Figure 10 is a schematic diagram of material cutting by a wire saw. The center of the cross-section of the wire is the origin *O* of the coordinate system. The reverse direction of the workpiece feed speed is the *y*-axis, the reciprocating motion direction of the wire saw is the *z*-axis, and the *x*-axis is perpendicular to the *y*-axis and the *z*-axis. The shaded area indicates the removed material. If the time between the abrasive entering the workpiece and exiting it is Δ*t*, then the feed distance of the workpiece during this time is Δ*t*·*v*_w_.

Take a infinitesimal angle d*θ* at the *O*-*xy* plane angle. From the geometric relationship in the figure, it can be seen that the infinitesimal angle corresponding to the micro-arc segment d*s* can be expressed as

(18) ds=Rdθ

where *R* is the radius of the wire.

Due to the high speed of the wire saw and the small size of the workpiece, the feed volume of the wire saw within Δ*t* is extremely small. The micro-element region can be regarded as a rectangle, and the area of this region is

(19) $$dS_{n}=v_{w}\cdot \Delta t\sin\theta ds$$

When the workpiece length is *L*, the workpiece removal volume corresponding to this micro-element region is

(20) $$V_{w}=v_{w}\cdot \Delta t\sin\theta RLd\theta$$

The number of abrasives *N* involved in cutting in the microelement region can be expressed as

(21) N=C·Lds=CLR·dθ

where *C* represents the abrasive density.

According to Equations (20) and (21), the volume of material removed by a single abrasive in this micro-element region can be obtained as

(22) $$V_{\mathrm{sd}}=\frac{V_{w}}{N}=\frac{v_{w}\cdot \Delta t\sin\theta }{C}$$

The abrasives on both sides of the wire saw mostly remove the material in a ductile mode. Assuming the abrasives are conical, the cross-section of the material removed is approximately triangular, with an area of

(23) $$A_{t}={h_{u}}^{2}\tan\beta$$

According to Equations (22) and (23), the depth at which a single abrasive penetrates the material can be calculated as

(24) $$h_{u}=\sqrt{\frac{v_{w}L\sin\theta }{v_{s}Cl_{u}\tan\beta }}$$

where *l*_u_ represents the trajectory length of the abrasives with UADWS.

Without applying ultrasonic vibration to the wire saw, that is, under the cutting conditions of DWS, the depth to which a single abrasive cuts into the material is

(25) $$h_{c}=\sqrt{\frac{v_{w}L\sin\theta }{v_{s}Cl_{c}\tan\beta }}$$

where *l*_c_ represents the trajectory length of the abrasives with DWS.

By comparing Equations (24) with (25), it can be seen from the discussion results in Section 2.2.1 that when the processing parameters are the same, the length *l*_u_ of the motion trajectory of the abrasives in the processing area under UADWS is greater than the length *l*_c_ of the motion trajectory of the abrasives in the processing area under the DWS. Therefore, the cutting depth of the abrasives after the wire saw is cut by ultrasonic vibration is less than that of the abrasives cut by the ordinary wire saw. This will make it easier for the abrasives to remove the material in a ductile mode, which is of great significance for reducing the sawing force and improving the surface quality of the slices.

## 3. Materials and Methods

**Figure 11:**
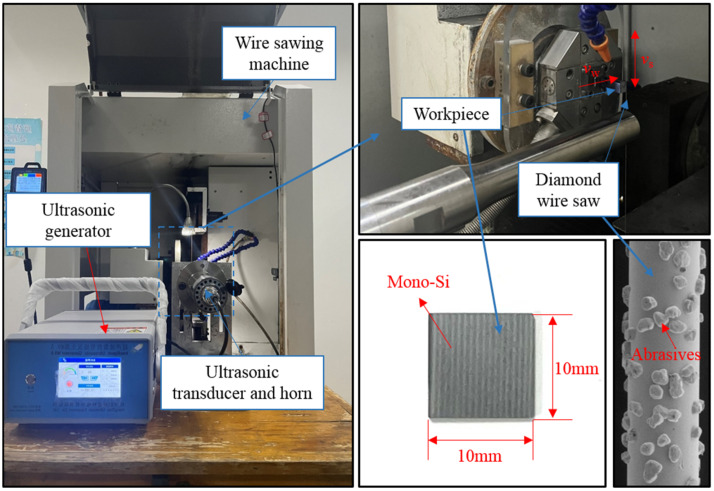
Experimental equipment, monocrystalline silicon slices, and diamond wire saw.

The experimental equipment is a diamond single-wire reciprocating cutting machine (SH300, Guangzhou Shenghai Electronic Technology Co., Ltd., Guangzhou, China) with an ultrasonic system. The ultrasonic system consists of an ultrasonic generator, an amplitude transformer, a horn and a holder. Monocrystalline silicon was cut using an electroplated diamond wire saw. The wire saw used in this experiment was 70 m in length and 220 μm in diameter, consisting of a wire substrate, a nickel plating layer, and randomly distributed diamond abrasives. A square monocrystalline silicon specimen with a size of 10 mm × 10 mm was selected as the material for the experiment. The experimental equipment and materials are shown in Figure 11.

**Figure 12:**
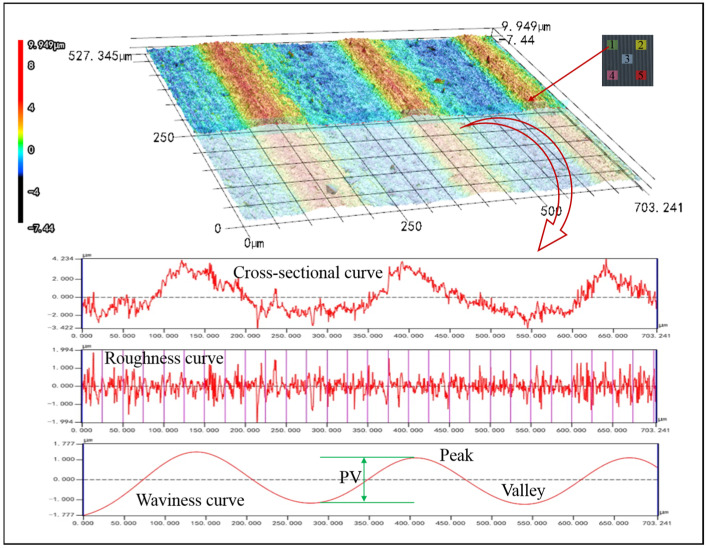
Schematic diagram of the measurement of slices.

In order to compare the differences in PV value, surface roughness, and surface morphology between mono-Si slices cut by UADWS and DWS, and to analyze the mechanism by which UADWS improves the surface of slices from the perspective of the microscopic movement of abrasives, a comparative experiment was conducted between DWS (*v*_w_ = 0.4 mm/min, *v*_s_ = 1200 m/min) and UADWS (*v*_w_ = 0.4 mm/min, *v*_s_ = 1200 m/min, *A* = 18 μm). After the experiment was completed, the surface of the sections was measured by a laser confocal microscope VK-X200K (Keyence Corporation, Osaka, Japan). To ensure the measurement accuracy, five points were selected on each section surface for measurement and the average value was taken, as shown in Figure 12.

## 4. Results and Discussion

### 4.1. The Experimental Results of Surface Roughness and PV Value

**Figure 13:**
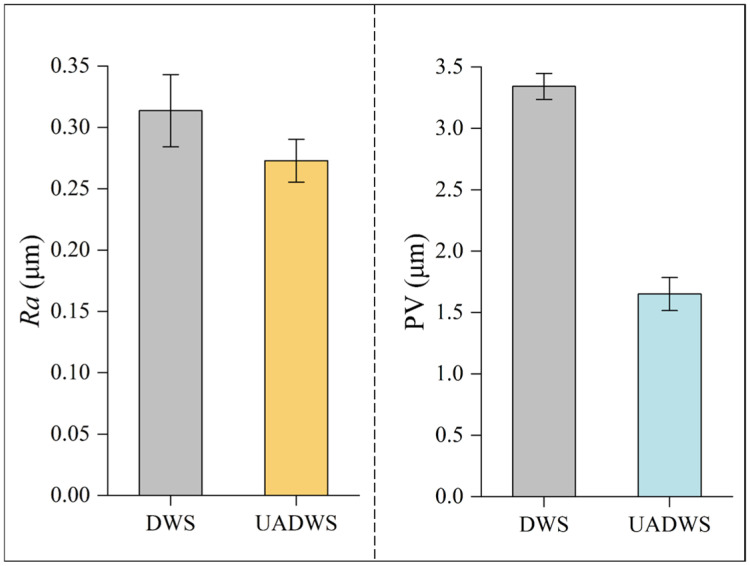
The surface roughness and PV value of slices under different processing methods.

Figure 13 shows the surface roughness and wire marks PV values of mono-Si slices cut by DWS and UADWS, respectively.

It can be seen from the experimental results that when ultrasonic vibration is applied to the wire saw, both the surface roughness and PV value of the mono-Si are reduced. The following analysis will delve into the reasons behind the improvement in slice surface quality due to ultrasonic vibration, based on the microscopic motion of abrasives and in conjunction with the experimental findings.

### 4.2. Analysis of the Surface Morphology of Mono-Si

**Figure 14:**
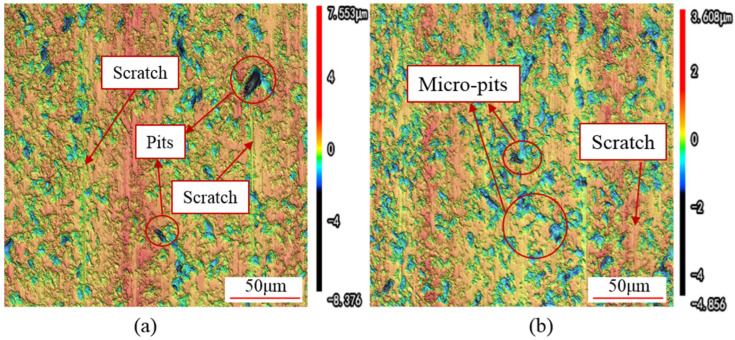
Surface morphologies of mono-Si slices: (**a**) DWS; (**b**) UADWS.

To observe the surface morphology of the slices in greater detail, a confocal microscope with a 1000× magnification was used to examine the slice surfaces, as shown in Figure 14.

Figure 14a depicts the surface morphology of a slice cut by DWS. As can be observed from the figure, the surface of the slice is distributed with large-sized pits. Additionally, relatively intact abrasive scratches can be seen, running parallel to each other. Due to the presence of protruding peaks and large-sized pits on the slice surface, the surface flatness of the slice is poor.

Figure 14b illustrates the surface morphology of a slice cut by UADWS. After applying ultrasonic vibration, the slice surface is uniformly distributed with a large number of micro-pits, which are very small in size. Moreover, it is difficult to observe complete scratches left by abrasives. Compared to slices cut by DWS, the surface of the slice obtained through UADWS has a greater number of pits, but these pits are smaller in size and uniformly distributed, resulting in a higher surface flatness. This makes it easier to remove the defects caused by the micro-pits and the surface flatness issues during subsequent processing. In contrast, DWS results in larger and deeper pits, necessitating the removal of more material in subsequent steps to eliminate the adverse effects caused by these large pits.

As can be inferred from the analysis of the abrasives’ motion trajectory in Section 2, there are notable differences in the trajectories of abrasives within the processing area between DWS and UADWS. When using DWS, the scratching trajectories of abrasives in the processing area approximate straight lines, with insignificant cross-interaction between the trajectories. Consequently, relatively straight scratches can be observed. Furthermore, as indicated by Equations (24) and (25), during DWS, the cutting depth of the abrasives is relatively large, resulting in a significant sawing force exerted by the wire saw [29,30]. This makes the material more prone to brittle removal, with lateral cracks continuously propagating and extending to the surface, leading to large-scale spalling of the material and the formation of blocky pits.

After applying ultrasonic vibration, the trajectories of abrasives on both sides of the kerf, which eventually form the surface area of the slice, approximate sinusoidal curves. The mutual scratching and abrading effects between different abrasive trajectories make it difficult for complete scratches to form on the final surface morphology. During the processing process, these scratches are interrupted by a large number of dot-like pits. The reason for the appearance of micro-pits is that when ultrasonic vibration is applied to the wire saw, the wire saw exerts a certain impact force on the slice surface. This energy causes the abrasives to leave a large number of micro-cracks on the slice surface. When subsequent abrasives cut through these micro-cracked areas, the force applied to the material is relatively small, making it easy for the material to peel off from the surface. The extent of lateral crack propagation is minimal, resulting in the formation of micro-pits. The higher surface flatness of the slice is also attributed to the ultrasonic-assisted machining process, during which large-sized pits do not appear on the surface. Moreover, the repeated scraping action of subsequent abrasives on the already-machined surface removes any remaining material that was not fully removed, contributing to the improved surface flatness.

### 4.3. Analysis of Surface Wire Marks of Mono-Si

**Figure 15:**
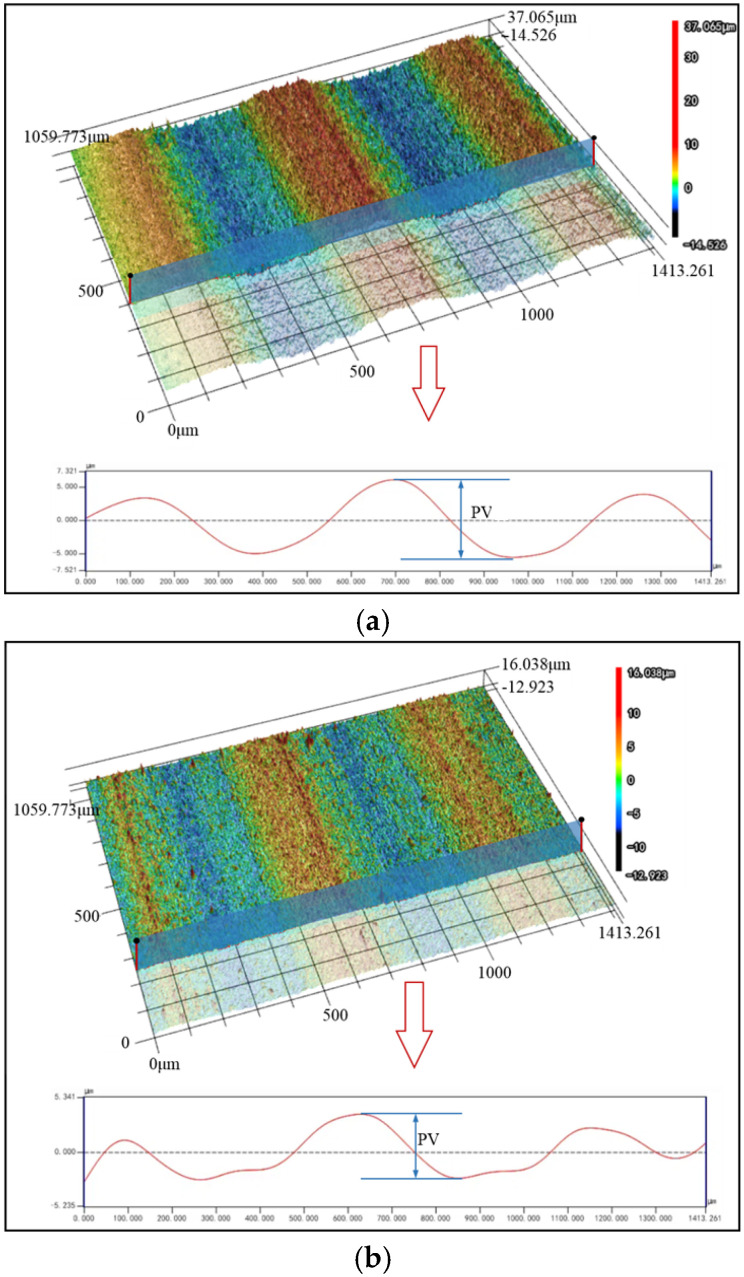
Wire marks on the surface of mono-Si slices: (**a**) DWS; (**b**) UADWS.

Since wire marks require a larger observation scale, a 200× magnification was used to observe the wire marks on the surface of slices. The 3D surface maps and the wire marks on the slices cut by DWS and UADWS are shown in Figure 15.

It can be observed that different sawing techniques have a significant impact on the wire marks on the surface of slices. As can be seen from Figure 14a, when DWS is employed, the peaks and valleys of the wire marks on the slice surface exhibit a relatively regular undulating pattern with the PV values being close in each cycle. Upon observing Figure 14b, it can be noted that when UADWS is utilized, since the processing parameters remain unchanged, the macroscopic undulation pattern and periodicity of the peaks and valleys of the wire marks on the slice surface are similar to those observed in DWS. However, when examining the details of each peak and valley, it is evident that the bottoms of the peaks and valley are partially trimmed. Compared to conventional sawing, UADWS removes material more thoroughly. This reduces the height difference between the peaks and valleys in each cycle, thereby decreasing the PV value.

**Figure 16:**
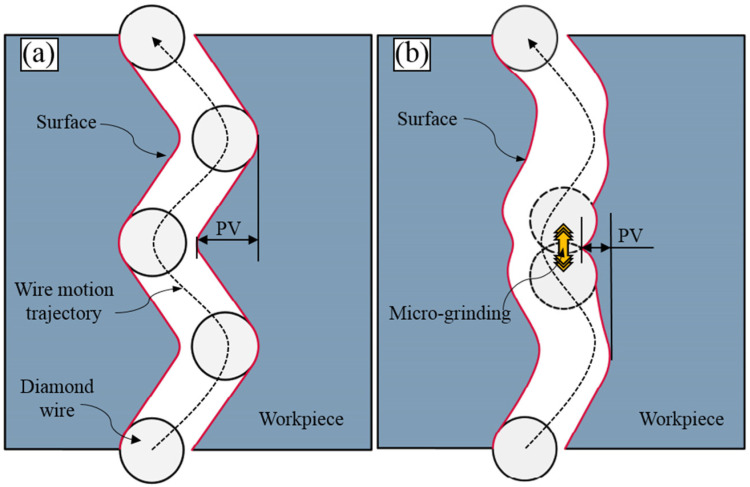
Schematic diagram of the formation mechanism of wire marks: (**a**) DWS; (**b**) UADWS.

Due to the reciprocating motion of the wire saw in the cutting machine, the oscillating phenomenon of the wire saw on both sides within the guide wheel groove causes it to produce periodic left-right swaying inside the kerf, resulting in the formation of wire mark patterns on the slice surface, as shown in Figure 16a.

Combining this with the analysis of abrasive trajectories, as illustrated in Figure 16b, the reason UADWS can reduce the PV value is that when the wire saw is positioned on one side of the kerf for cutting, the high-frequency ultrasonic vibration causes the wire saw to rapidly oscillate slightly in the vertical direction while moving axially. The diamond abrasives bonded to the wire matrix similarly perform rapid further material removal on the surface, exerting a minor grinding effect on the peaks and valleys of the emerging wire marks, effectively trimming the tips of these peaks and valleys. Consequently, this reduces the PV value of the wire marks and mitigates the issue of wire marks on the slice surface.

Therefore, when the UADWS technology is employed for material sawing, it alters the microscopic motion state of the abrasives. This leads to enhanced cross-interference effects among different abrasives, reducing the depth at which the abrasives penetrate the material and thereby minimizing surface damage on the slice. Moreover, the vibratory action of the abrasives performs micro-grinding on the slice surface, further mitigating the impact of wire marks. Consequently, compared to DWS, UADWS offers certain advantages in improving the surface quality of slices.

## 5. Conclusions

In this paper, a model for the motion trajectory of abrasives in UADWS was established. The cutting depth and cutting arc length of the abrasives were further calculated. Combining these calculations with experimental results, the reasons for the improved surface quality of slices after the composite ultrasonic vibration of the wire saw were analyzed from the microscopic perspective of abrasive motion. The main conclusions are as follows:

(1) When ultrasonic vibration is applied to the wire saw, the motion trajectory of a single abrasive changes from a straight line to an approximately sinusoidal curve. When processing is conducted at a higher wire speed, the number of vibrations of the abrasive within the processing area decreases, which, to a certain extent, weakens the effect of the ultrasonic vibration. Moreover, during ultrasonic-assisted sawing, the abrasive has a longer cutting arc length and a lower cutting depth, which has a beneficial impact on the surface quality of the slice.
(2) When using UADWS for sawing, abrasives at different positions on the wire saw surface experience various types of cross-interference. When the spacing between abrasives, Δ*L*, is not equal to the wavelength, *λ*, there is a significant degree of interference between the trajectories of the abrasives, which can enhance the material removal rate and eliminate any residual peaks on the material surface that were not fully removed. When Δ*L* is equal to *λ*, the trajectories of the abrasives approximately coincide, which, to a certain extent, reduces the material removal rate.
(3) Compared with DWS, the surface roughness *Ra* of the mono-Si slices obtained by UADWS is smaller, at 0.27 μm, while that of DWS is 0.31 μm. The surface morphology has small pit sizes and short scratches. Due to the micro-vibration effect of the abrasive grains, the PV value of the wire marks on the slice surface is lower, at 1.65 μm, while that of DWS is 3.34 μm. The surface pits of the slices cut with DWS are larger in size, but the number of large pits is smaller, and the scratches are parallel to each other. The line marks on the surface of the slice undulate regularly.

The conclusion of this study clarifies the advantages of UADWS cutting processing from the mechanistic perspective. On this basis, theoretical modeling of the sawing force and surface quality of UADWS can be further carried out in the future and has certain reference significance for the in-depth study of UADWS.

## Data Availability

Data are contained within the article.
